# Long Noncoding RNA MLK7-AS1 Promotes Non-Small-Cell Lung Cancer Migration and Invasion *via* the miR-375-3p/YWHAZ Axis

**DOI:** 10.3389/fonc.2021.626036

**Published:** 2021-04-22

**Authors:** Jingzhou Jia, Jiwei Sun, Wenbo Wang, Hongmei Yong

**Affiliations:** ^1^ Department of Thoracic Surgery, Henan Provincial Chest Hospital, Zhengzhou, China; ^2^ Department of Oncology, The Affiliated Huai’an Hospital of Xuzhou Medical University and The Second People’s Hospital of Huai’an, Huaian, China

**Keywords:** non-small-cell lung cancer, noncoding RNA, MLK7-AS1, miR-375-3p, YWHAZ

## Abstract

Long noncoding RNAs act essential regulators in lung cancer tumorigenesis. Our research aimed to investigate the potential function and molecular mechanisms of MLK7-AS1 in NSCLC (non-small-cell lung cancer). QRT-PCR results indicated that the MLK7-AS1 expression level was upregulated in NSCLC cells and tissues. MLK7-AS1 strengthened cell migration and invasion in H1299 and A549 cells. Luciferase reporter assay found that MLK7-AS1 functioned as an endogenous sponge for miR-375-3p. Transwell assay results showed that miR-375-3p suppressed cell migration and invasion in H1299 and A549 cells. YWHAZ was confirmed as a target gene of miR-375-3p by Targetscan. YWHAZ overexpression promoted the invasion of H1299 and A549 cells. MLK7-AS1 upregulated YWHAZ expression and enhanced H1299 and A549 cell invasion by sponging miR-375-3p. MLK7-AS1 improved the metastasis ability of A549 *in vivo*. In conclusion, MLK7-AS1 was identified as a novel oncogenic RNA in NSCLC and can function as a potential therapeutic target for NSCLC treatment.

## Introduction

NSCLC is the main cause of thoracic neoplasms in the world (1, 2). Unfortunately, the rate of lung cancer diagnoses is increasing steadily in China (3). Considerable advances in operation and chemoradiotherapy have been achieved, but a high frequency of recurrence in patients with lung cancer remains a problem in lung cancer treatment (4). Understanding the mechanisms of NSCLC development is the cornerstone of solving clinical questions.

Long noncoding RNAs (lncRNAs) are noncoding RNA molecules longer than 200 nucleotides (5, 6). LINC01296 promotes proliferation in NSCLC viamiR-5095/Wnt axis (7). FLVCR1-AS1 downregulation suppresses cell invasion in NSCLC through Wnt/β-catenin signaling pathway (8). LINC00163 overexpression inhibits lung cancer progression by transcriptionally upregulating TCF21 expression (9). SLCO4A1-AS1 accelerates the colorectal cancer development through Wnt signaling pathway (10). lncRNA PVT1 facilitates the tumor progression in gallbladder cancer *via* the miR-143/HK2 axis (11). MLK7 antisense RNA 1 (MLK7-AS1) was previously identified as an oncogene in several tumors. For example, MLK7−AS1 can enhance ovarian cancer cells invasion by upregulating the expression of YAP1 (12). However, the role of MLK7−AS1 in NSCLC is largely unclarified.

MicroRNAs (miRNAs), small noncoding RNAs of 20–25 nucleotides in length, regulate the expression of downstream targets through post-transcriptional modulation (13, 14). Competing endogenous RNAs (ceRNA) is a vital mechanism regulating the progression of various cancers. In-depth studies have demonstrated that lncRNAs acted as a vital regulatory role in malignancies as competing endogenous RNAs. FLVCR1-AS1 enhances gastric cancer tumorigenesis by sponging miR-155 and targeting c-Myc (15). lncRNA SNHG16 accelerates the cancer cells migration and invasion abilities through sponging miR-520d-3p and targeting STAT3 in hemangioma (16). CeRNA network is the vital mechanism of tumor development (17). lncRNA ZEB1-AS1 promotes TGF-β1-induced invasion of bladder tumor cells *via* targeting the miR-200b/FSCN1 pathway (18). Thus, exploring the roles of lncRNAs in NSCLC can help scholars understand potential mechanisms from a new perspective.

In this study, we found MLK7−AS1 was upregulated in NSCLC, which indicated MLK7−AS1 was a favorable factor for NSCLC. To further demonstrate the role of MLK7−AS1 and the underlying mechanism in NSCLC, NSCLC cell lines and *in vivo* models was used, which would contribute to the understanding of the development and progression of NSCLC, thus could provide potential therapeutic target for NSCLC.

## Material and Methods

### Cell Culture

Three NSCLC lines (i.e., H1299, A549, and H1650) and the normal cell line BEAS-2B were cultured in an incubator (37°C, 5% CO_2_) and in RPMI1640 (Gibco BRL, Gaithersburg, MD, USA) supplemented with 10% FBS (Gibco BRL, Gaithersburg, MD, USA).

### Transwell Migration and Invasion Assay

Transwell migration and invasion assay was performed in accordance with previously described method (19). For the Transwell migration assays, the transfected H1299 and A549 (N=5.5×10^3^) cells were plated in top chambers with a noncoated membrane (Invitrogen, Carlsbad, CA). For the invasion assays, the transfected H1299 and A549 (N=11×10^3^) cells were plated in top chambers with a coated membrane. The number of invading cells was counted after fixed with 4% paraformaldehyde (Beyotime, Shanghai, China).

### Cell Transfection and Lentivirus Production

The sequence of YWHAZ was cloned into the pcDNA3.1 vector and the empty vector acted as a negative control. The lentiviral vector for MLK7−AS1 was purchased from Kaiji Gene (Shanghai, China). mimic/inhibitor-miR-375-3p and mimic/inhibitor-NC were purchased from RiboBio (China, Guangzhou). Lipofectamine 2000 (Invitrogen, Carlsbad, CA) was utilized for transfection in accordance with the manufacturer’s instructions.

### Nuclear and Cytoplasmic RNA Isolation

Nuclear and cytoplasmic RNA was isolated in accordance with previously described methods (20, 21). The cytoplasm and nuclear RNAs of NSCLC cells were separated and extracted using a nuclear and cytoplasmic RNA purification kit (Invitrogen, Carlsbad, CA). qPCR assay was performed to detect the isolated RNA.

### Quantitative Reverse Transcription PCR (qRT-PCR)

The RNA of NSCLC cells was isolated using TRIzol reagent (US, Life Technologies, USA). SYBR Green qRT-PCR on an ABI7300 real-time PCR machine was used to measure the expression levels of mRNAs. YWHAZ and GAPDH expression levels were examined using the following specific primers:

5′CCTGCATGAAGTCTGTAACTGAG3′,
5′GACCTACGGGCTCCTACAACA3′,
5′GGAGCGAGATCCCTCCAAAAT3′, and
5′GGCTGTTGTCATACTTCTCATGG3′.

### Western Blot Analysis

The protein (15–20 mg) extracted from cells was used for Western blot analysis. The antibodies utilized in this study included anti-YWHAZ (1:1000; Cell Signaling Technology, USA) and GAPDH (1:1000; Cell Signaling Technology, USA). GAPDH (1:2000; Cell Signaling Technology, USA) was used as a loading control.

### Luciferase Reporter Assay

Luciferase reporter assay was performed in accordance with previously described procedures (22). Wt-pmirGLO-, MLK7−AS1, wt-pmirGLO-YWHAZ, and their corresponding mutated vectors were constructed. Wt-pmirGLO or mut-pmirGLO was cotransfected into cells with miR-375-3p inhibitor or miR-375-3p mimic by using Lipofectamine 2000. Luciferase activity was detected 48h after transfection.

### *In Vivo* Study

Six-week-old female nude mice were obtained from Medical Center of Yangzhou University. (Yangzhou, China). The A549 cell line stably overexpressing MLK7-AS1 was established. A lung metastasis mice model was also established with the intra-splenic injection of 5×10^6^ stably overexpressing MLK7-AS1 or LV-NC cells. After 24 days, lung colonization capacity was evaluated. The number of lung metastatic foci was counted *via* H&E staining. This work was approved by the Medical Ethics Committee of Henan Provincial Chest Hospital.

### Statistical Analysis

GraphPad Prism 5.0 and SPSS 13.0 were used to analyze data. Statistical data were expressed as mean ± standard deviation. Differences were considered significant at P<0.05.

## Results

### MLK7-AS1 Promoted the Migration and Invasion of NSCLC cells

**Figure 1:**
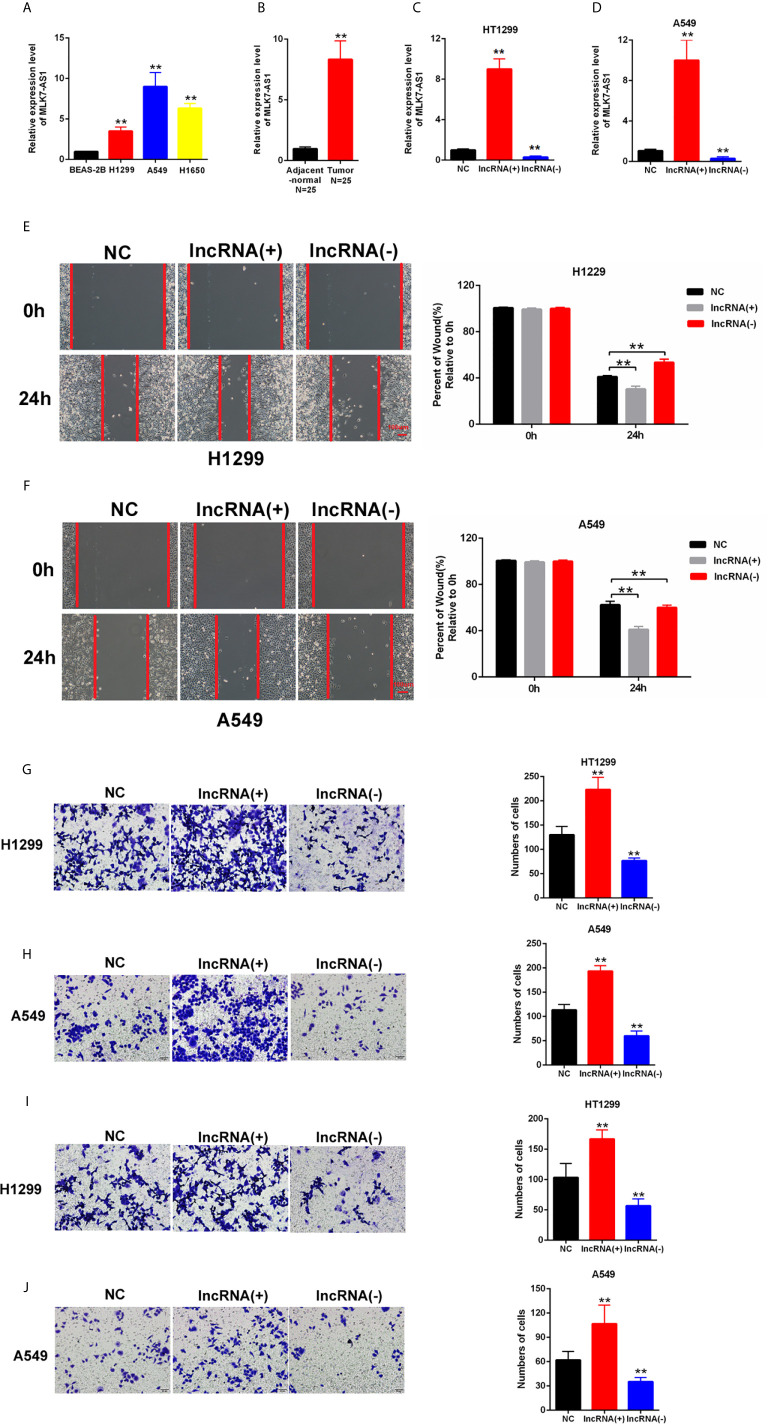
Upregulated MLK7-AS1 enhanced the migration and invasion of NSCLC cells. **(A)** MLK7-AS1 expression level was upregulated in the NSCLC cell lines compared with that in the normal line. **(B)** MLK7-AS1 expression level was upregulated in NSCLC tissues compared with that in tumor-adjacent normal pairs (*N*=25). **(C, D)** LV-MLK7-AS1 and sh-MLK7-AS1 efficiency was measured through qRT-PCR. **(E, F)** Effect of MLK7-AS1 on migration detected through wound-healing assays. LV-MLK7-AS1 promoted cell migration, whereas sh-MLK7-AS1 inhibited cell migration in H1229 and A549. **(G, H)** Effect of MLK7-AS1 on migration detected through Transwell assays. LV-MLK7-AS1 strengthened cell migration, whereas sh-MLK7-AS1 suppressed cell migration in H1229 and A549. **(I, J)** Effect of MLK7-AS1 on invasion detected through Transwell assays. LV-MLK7-AS1 increased cell invasion, but sh-MLK7-AS1 decreased cell invasion in H1229 and A549. Data indicate the mean ± SD, n = 3. **P < 0.01 vs. control.

MLK7-AS1 expression was detected by qPCR in NSCLC cell lines, namely, H1299, A549, H1650 compared with normal human bronchial epithelium BEAS-2B. As shown in **Figure 1A**. The expression level of MLK7-AS1 was upregulated NSCLC cell lines. Moreover, the expression level of MLK7-AS1 was upregulated in NSCLC tissues than in adjacent tissues (N=25) (**Figure 1B**). To explore the biological functions of MLK7-AS1, overexpression (LV-MLK7-AS1) and knockdown (sh-MLK7-AS1) assays were performed in H1299 and A549 cells. The efficiency of LV-MLK7-AS1 and sh-MLK7-AS1 was determined using qRT-PCR (**Figures 1C, D**). Wound-healing assays results indicated that MLK7-AS1 overexpression promoted the cells migration and silencing of MLK7-AS1 weakened the cells migration in H1299 and A549 (**Figures 1E, F**). Moreover, transwell assays indicated that the cell migration in H1299 and A549 was strengthened by MLK7-AS1 upregulation but was weakened by MLK7-AS1 downregulation (**Figures 1G, H**). The cell invasion in H1299 and A549 was promoted by MLK7-AS1 overexpression but was suppressed by MLK7-AS1 knockout knockdown (**Figures 1I, J**). These results demonstrated that the MLK7-AS1 strengthened the NSCLC cells migration and invasion.

### miR-375-3p Was Predicted as a Direct Target of MLK7-AS1

**Figure 2:**
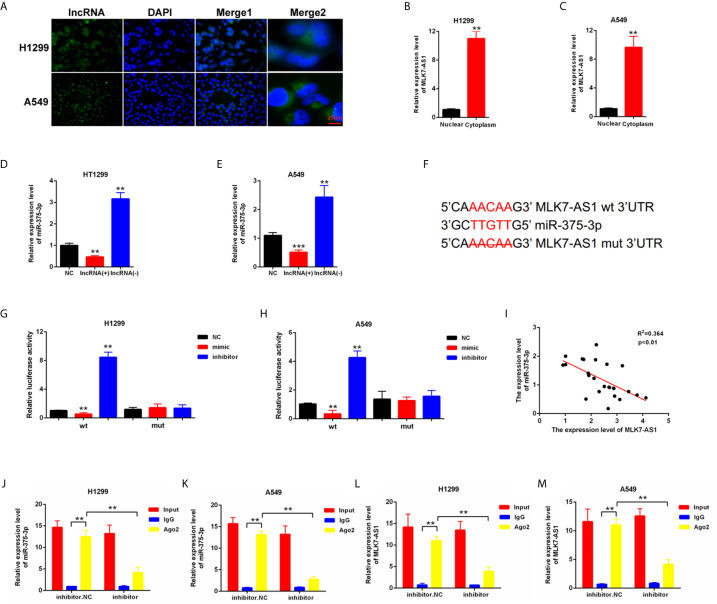
miR-375-3p was predicted as a direct target of MLK7-AS1. **(A)** FISH assays were conducted to detect the location of MLK7-AS1 in H1299 and A549. MLK7-AS1 was mainly localized in the cytoplasm. **(B, C)** qRT-PCR was performed to determine the location of MLK7-AS1 in H1299 and A549. MLK7-AS1 expression level was higher in the cytoplasm than in nucleus. **(D, E)** qRT-PCR results showed that MLK7-AS1 upregulation decreased the miR-375-3p expression level, whereas MLK7-AS1 downregulation increased the miR-375-3p expression level. **(F)** Direct binding sites between MLK7-AS1 and miR-375-3p were presented. **(G, H)** Luciferase reporter assay was performed to confirm the direct binding relationship between MLK7-AS1 and miR-375-3p. **(I)** The regression analysis of correlation between the expression of MLK7-AS1 and miR-375-3p in lung cancer tissues (n=22). **(J–M)** After RIP assay in H1299 or A549, the levels of MLK7-AS1 and miR-375-3p were respectively quantified by RT-qPCR. Data indicate the mean ± SD, n = 3. **P < 0.01, ***P < 0.001 vs. control.

To explore whether competing endogenous RNA (ceRNA) was involved in the regulation of MLK7-AS1 in NSCLC, FISH assays were performed in H1299 and A549. MLK7-AS1 mainly localized in the cytoplasm (**Figure 2A**). QRT-PCR also indicated that MLK7-AS1 mainly expressed in the cytoplasm of H1299 and A549 cells (**Figures 2B, C**). The potential target miRNAs for MLK7-AS1 were predicted using lncBase (lncBase/Experimental/?r=lncBase) and StarBase v2.0 (http://starbase.sysu.edu.cn/). Among many candidates, miR-375-3p could act as a tumor suppressor in several tumors (23). Thus, miR-375-3p was identified as a prior candidate for MLK7-AS1. LV-MLK7-AS1 and sh-MLK7-AS1 were transfected in NSCLC cells to explore the relationship between MLK7-AS1 and miR-375-3p, which revealed that LV-MLK7-AS1 downregulated the miR-375-3p expression, whereas sh-MLK7-AS1 upregulated the miR-375-3p expression in H1299 and A549 cells **(** **Figures 2D, E** **)**. StarBase v2.0 was used to predict the specific binding site between MLK7-AS1 and miR-375-3p, which result was shown in **Figure 2F**. Moreover, dual-luciferase reporter assays were performed in H1299 and A549. As shown in **Figures 2G, H**, luciferase activity could be inhibited by mimic-miR-375-3p but could be promoted by inhibitor-miR-375-3p in WT-MLK7-AS1 reporter. However, the mutant-type reporter gene (MT-MLK7-AS1 reporter) was not inhibited or increased in the luciferase activity by mimic-or inhibitor-miR-375-3p. In addition, we examined the expression levels of miR-375-3p in lung cancer tissues (n=22), and made the correlation analysis with the expression levels of MLK7-AS1 **(** **Figure 2I** **)**. We found that the expression levels of miR-375-3p were negatively correlated with the expression levels of MLK7-AS1. A RIP assay was performed to examine whether MLK7-AS1 and miR-375-3p are in the same RISC complex. Then, RIP assays indicated that MLK7-AS1 and miR-375-3p were enriched in Ago2 compared with control IgG. Subsequently, miR-375-3p got a significant enrichment in the MLK7-AS1 pull down pellets compared with control IgG. Moreover, after transfection with inhibitor-miR-20a-5p, the expression levels of MLK7-AS1 and miR-375-3p enriched in Ago2 were downregulated **(** **Figures 2J–M** **)**. Thus, miR-375-3p was a direct target of MLK7-AS1.

### miR-375-3p Suppressed the Migration and Invasion of NSCLC Cells

**Figure 3:**
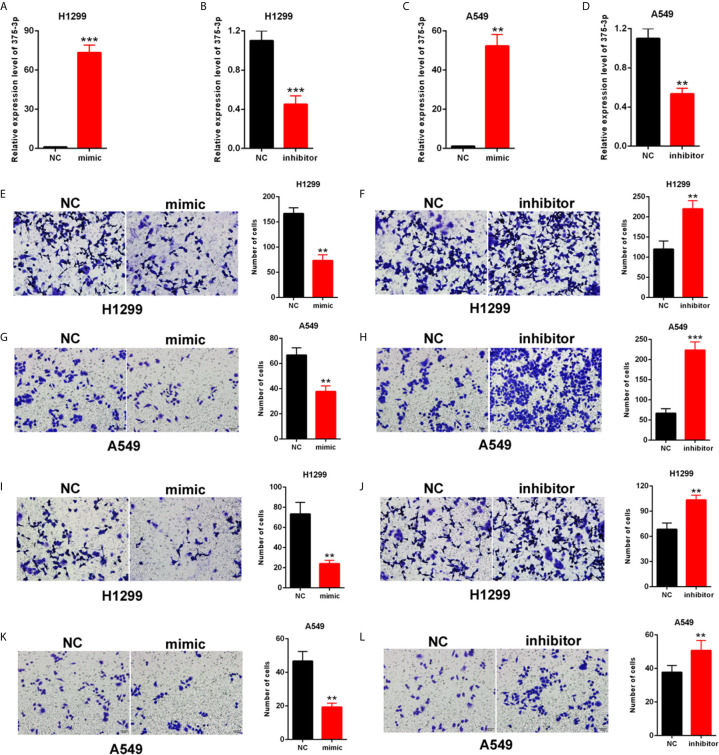
Effects of miR-375-3p on the migration and invasion of NSCLC cells. **(A, B)** Efficiency of mimic/inhibitor-miR-375-3p was determined through qPCR in H1299. **(C, D)** Efficiency of mimic/inhibitor-miR-375-3p was determined through qRT-PCR in A549. **(E–H)** Effect of miR-375-3p on migration was detected through Transwell assays in H1299 and A549. Cell migration was promoted by miR-375-3p-mimic but was suppressed by miR-375-3p-inhibitor. **(I–L)** Effects of miR-375-3p on invasion detected through Transwell assays in H1299 and A549. Cell invasion was enhanced by miR-375-3p-mimic but was suppressed by miR-375-3p-inhibitor in H1299 and A549. Data indicate the mean ± SD, n = 3. **P < 0.01, ***P < 0.001 vs. control.

H1299 and A549 were transfected with mimic/inhibitor of miR-375-3p to investigate the role of miR-375-3p in NSCLC cells. The efficiency of mimic-miR-375-3p and inhibitor-miR-375-3p was measured through qRT-PCR **(** **Figures 3A–D** **)**. The cell migration was inhibited by mimic-miR-375-3p and was enhanced by inhibitor-miR-375-3p in H1299 and A549 **(** **Figures 3E–H** **)**. The cell invasion was inhibited by mimic-miR-375-3p and was enhanced by inhibitor-miR-375-3p in H1299 and A549 **(** **Figures 3I–L** **)**. Thus, miR-375-3p suppressed cell migration and invasion in H1299 and A549.

### YWHAZ Was a Direct Target Gene of miR-375-3p

**Figure 4:**
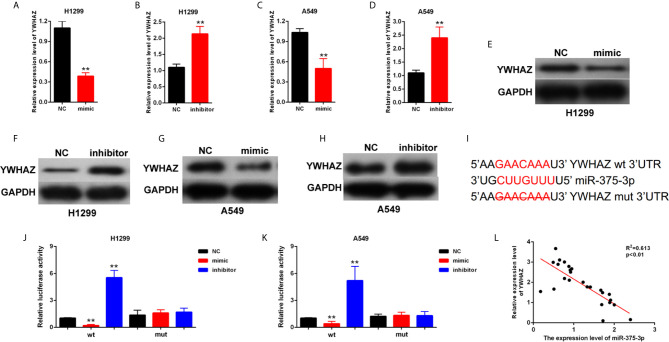
YWHAZ was a direct target gene of miR-375-3p. **(A–D)** qRT-PCR results indicated that the expression level of YWHAZ was decreased by miR-375-3p-mimic but was increased by miR-375-3p-inhibitor. **(E–H)** Western blot results revealed that the expression level of YWHAZ was increased by miR-375-3p overexpression but was decreased by miR-375-3p. **(I)** Direct binding sites between miR-375-3p and YWHAZ were presented. **(J, K)** Luciferase reporter assays were performed in H1299 and A549. Direct binding relationship between miR-375-3p and YWHAZ was confirmed. **(L)** The regression analysis of correlation between the expression of miR-375-3p and YWHAZ in lung cancer tissues (n=22). Data indicate the mean ± SD, n = 3. **P < 0.01 vs. control.

The potential target genes of miR-375-3p were predicted by StarBase v2.0. Among many candidates, YWHAZ was identified as an oncogene in several tumors (24, 25). Thus, YWHAZ was chosen as a prior candidate for miR-375-3p. As shown in **Figures 4A–H**, results indicated that the mRNA and protein expression level of YWHAZ was downregulated after transfection with mimic-miR-375-3p and was upregulated after transfection with inhibitor-miR-375-3p. The analysis through StarBase v2.0 revealed the direct binding sites between miR-375-3p and YWHAZ **(** **Figure 4I** **)**. Moreover, dual-luciferase reporter experiments were performed in H1299 and A549. The luciferase activity was inhibited by mimic-miR-375-3p and was enhanced by inhibitor-miR-375-3p in WT-YWHAZ reporter. However, the mutant-type reporter gene (MT- YWHAZ reporter) was not inhibited or enhanced by mimic-miR-375-3p or inhibitor-miR-375-3p **(** **Figures 4J, K** **)**. We found that the expression levels of YWHAZ were negatively correlated with the expression levels of miR-375-3p in lung cancer tissues (n=22, **Figure 4L**). As such, YWHAZ was a direct target gene of miR-375-3p.

### MLK7-AS1 Upregulated the YWHAZ Expression Level and Promoted Invasion by Sponging miR-375-3p

**Figure 5:**
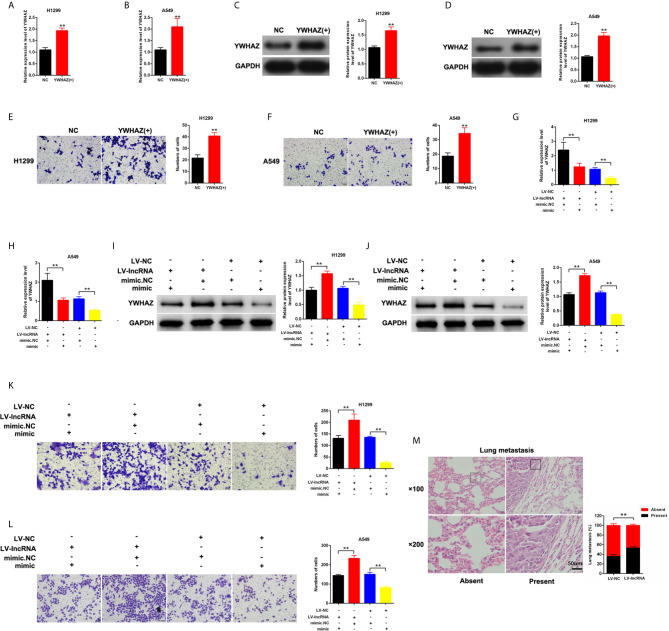
MLK7-AS1 upregulated YWHAZ expression and promoted the invasion by acting as miR-375-3p sponge. **(A, B)** Efficiency of pcDNA3.1-YWHAZ was determined through qPCR in H1229 and A549 cells. **(C, D)** Western blot results showed that YWHAZ gene was successfully overexpressed after transfection with pcDNA3.1-YWHAZ. **(E, F)** Overexpression of YWHAZ enhanced cell invasion in H1229 and A549. **(G, H)** mRNA expression level of YWHAZ was detected through qPCR. H1299 and A549 cells were transfected with LV-MLK7-AS1 or mimic-miR-375-3p. **(I, J)** Protein expression level of YWHAZ was detected by western blot. H1299 and A549 cells were transfected with LV-MLK7-AS1 or mimic-miR-375-3p. **(K, L)** Invasion was detected through Transwell assays. Cells were transfected with LV-MLK7-AS1 or mimic-miR-375-3p. **(M)** Typical images for the lung metastasis of a mouse model. The percentage of mice with or without metastatic nodules in the lungs was counted. Data indicate the mean ± SD, n = 3., **P < 0.01 vs. control.

YWHAZ was overexpressed in H1299 and A549 cells **(** **Figures 5A–D** **)**. We found that the upregulation of YWHAZ promoted cell invasion in H1299 and A549 cells **(** **Figures 5E, F** **)**. Restore experiments were performed in H1299 and A549, and the cells were cotransfected with mimic-miR-375-3p or LV-MLK7-AS1. miR-375-3p overexpression could restore the upregulation of YWHAZ in NSCLC cells after transfection with LV-MLK7-AS1 **(** **Figures 5G–J** **)**. mimic-miR-375-3p could restore the improvement of invasion ability in NSCLC cells after transfection with LV-MLK7-AS1 **(** **Figures 5K, L** **)**. Lastly, the stable MLK7-AS1-overexpression in A549 cell line was established. Lung metastasis models were established by inoculation of A549 cells. As shown in **Figure 5M**, MLK7-AS1 overexpression could enhance the metastasis ability of A549 cells. Thus, these results suggested that MLK7-AS1 upregulated the YWHAZ expression level and enhanced the invasion by acting as miR-375-3p sponge in NSCLC.

## Discussion

NSCLC is a highly aggressive tumor and has a poor five-year survival rate. Metastasis and recurrence are the important negative prognostic factors of NSCLC. Understanding the molecular mechanism of NSCLC development is helpful to address its poor survival rate. lncRNAs are important regulators of NSCLC progression. lncRNA NEF inhibits NSCLC proliferation by targeting glucose transport (26). LINC00702 inhibits tumor growth and invasion in NSCLC *via* the miR-510/PTEN axis (27). A novel lncRNA BC200 regulates the PI3K/AKT pathway and promoted the development of NSCLC (28).

In this study, MLK7-AS1 was identified as a vital regulator in NSCLC. Firstly, we found that MLK7-AS1 was upregulated in H1299, A549 and H1650 cell lines and NSCLC tissues, which suggested that MLK7-AS1 might participate in the progression of NSCLC. In order to study the role of MLK7-AS1 in NSCLC, overexpression and knockdown of MLK7-AS1 were performed. We found that LV-MLK7-AS1 strengthened the invasion of H1299 and A549, whereas, sh-MLK7-AS1 weakened the invasion of NSCLC cells. In-depth researches have proven that lncRNAs acted as an important regulatory role in malignancies as competing endogenous RNAs[14]. Then, miR-375-3p was identified as a direct target of MLK7-AS1. Previous studies suggested that miR-375-3p participates in tumor development. miR-375-3p may act as a tumor suppressor by targeting LAMC1 in HNSCC (29). However, the role of miR-375-3p in NSCLC is unknown.

Overexpression and knockdown assays were performed in NSCLC cells. Transwell assays indicated that the invasion of the NSCLC cells could be suppressed by mimic-miR-375-3p but could be enhanced by inhibitor-miR-375-3p. The binding between lncRNA and miRNA is according to bases of complementary matching principle (29). Then, dual-luciferase reporter assays were performed in H1299 and A549. miR-375-3p regulated the luciferase activity in WT-MLK7-AS1 reporter. But, the luciferace activity of the mutant-type reporter gene was not decreased or increased by miR-375-3p. Thus, miR-375-3p was a direct target of MLK7-AS1. By binding to the 3’UTR region of the coding gene, miRNAs downregulated the target genes expression (27). YWHAZ was predicted as a target gene for miR-375-3p. Results indicated that the mRNA and protein expression of YWHAZ were upregulated by mimic-miR-375-3p but was downregulated by inhibitor-miR-375-3p in H1299 and A549. Dual-luciferase reporter assays indicated that YWHAZ was a direct target gene for miR-375-3p. The role of YWHAZ in NSCLC was further investigated. YWHAZ enhances metastasis and is related to the poor survival in hepatocellular carcinoma (30). YWHAZ strengthens the gastric cancer cells growth ability by suppressing cell apoptosis and autophagy (24).

However, the role of YWHAZ in NSCLC is unclear. YWHAZ was overexpressed in NSCLS cells *via* pcDNA3.1-YWHAZ transfection. YWHAZ overexpression promoted the cell invasion in H1299 and A549. The effect of miR-375-3p in MLK7-AS1 function was investigated. The NSCLC cells were transfected with LV-MLK7-AS1 or mimic-miR-375-3p. Restore experiments confirmed that MLK7-AS1 promoted the cell invasion and upregulated the YWHAZ expression by sponging miR-375-3p. The effect of MLK7-AS1 was explored *in vivo*. A lung metastasis mouse model was established, and the MLK7-AS1 overexpression enhanced the metastasis ability of NSCLC cells *in vivo*.

## Conclusion

In summary, our study reported that MLK7-AS1 was upregulated in NSCLC and can promote cell invasion *in vitro* and *in vivo* through upregulating miR-375-3p/YWHAZ axis. MLK7-AS1 might act as a potential diagnostic biomarker and therapeutic target for NSCLC.

## Data Availability Statement

The original contributions presented in the study are included in the article/supplementary material. Further inquiries can be directed to the corresponding author.

## Ethics Statement

The animal study was reviewed and approved by Henan Provincial Chest Hospital.

## Author Contributions

WW and HY designed and revised the study. JJ performed the experiments and prepared the manuscript. JS and HY collected and analyzed the data. All authors contributed to the article and approved the submitted version.

## Conflict of Interest

The authors declare that the research was conducted in the absence of any commercial or financial relationships that could be construed as a potential conflict of interest.

## Abbreviations

qPCR, Quantitative reverse transcription polymerase chain reaction; lncRNAs, long noncoding RNAs; ceRNA, competing endogenous RNA; NSCLC, non-small-cell lung cancer.
